# Examining Land-Use/Land-Cover Change in the Lake Dianchi Watershed of the Yunnan-Guizhou Plateau of Southwest China with Remote Sensing and GIS Techniques: 1974-2008

**DOI:** 10.3390/ijerph9113843

**Published:** 2012-10-24

**Authors:** Yaolong Zhao, Ke Zhang, Yingchun Fu, Hong Zhang

**Affiliations:** 1 School of Geography, South China Normal University, Guangzhou 510631, China; Email: yaolong@scnu.edu.cn (Y.Z.); kezhang24@gmail.com (K.Z.); 2 School of Urban and Environmental Studies, Yunnan University of Finance and Economics, Kunming 650221, China; Email: zhanghong0957@sina.com

**Keywords:** Yunnan-Guizhou plateau, Lake Dianchi, land-use/land-cover change (LULCC), remote sensing, geographical information system (GIS), Landsat

## Abstract

Monitoring land-use/land-cover change (LULCC) and exploring its mechanisms are important processes in the environmental management of a lake watershed. The purpose of this study was to examine the spatiotemporal pattern of LULCC by using multi landscape metrics in the Lake Dianchi watershed, which is located in the Yunnan-Guizhou Plateau of Southwest China. Landsat images from the years 1974, 1988, 1998, and 2008 were analyzed using geographical information system (GIS) techniques. The results reveal that land-use/land-cover has changed greatly in the watershed since 1974. This change in land use structure was embodied in the rapid increase of developed areas with a relative change rate of up to 324.4%. The increase in developed areas mainly occurred in agricultural land, especially near the shores of Lake Dianchi. The spatial pattern and structure of the change was influenced by the urban sprawl of the city of Kunming. The urban sprawl took on the typical expansion mode of cyclic structures and a jigsaw pattern and expanded to the shore of Lake Dianchi. Agricultural land changed little with respect to the structure but changed greatly in the spatial pattern. The landscape in the watershed showed a trend of fragmentation with a complex boundary. The dynamics of land-use/land-cover in the watershed correlate with land-use policies and economic development in China.

## 1. Introduction

Surface water is an essential component of the natural environment. Because surface water is limited and affected by multiple factors, such as human activities and environmental changes, many lakes, rivers, and even seas throughout the World have been greatly polluted or overused, particularly in China since the well-known “open-door” policy and economic reform were adopted in 1978 [1,2]. Polluted surface water has had a strong impact on human health and quality of life [3]. Land-use/land-cover change (LULCC) is a dominant stressor in the water quality, ecosystem, and environmental deterioration of a watershed [4,5]. LULCC is threatening water quality throughout the World. Many scholars have quantitatively assessed the effects of LULCC on the water quality [6,7,8] and ecological environment [9,10,11] of watersheds with respect to surface runoff, land erosion, non-point source pollution, and sewage from urban areas [3,12,13] using remote sensing, geographical information systems (GIS), modeling, and sample analysis approaches. Therefore, it can be inferred that examining LULCC should be fundamental first step for improving the water quality and ecological environment of a watershed. 

Dianchi is one of the famous large lakes in China. It is located in the Yunnan-Guizhou Plateau of southwest China. The near shore area of Lake Dianchi has become one of the cradles of human civilization in the Yunnan-Guizhou Plateau and is one of the regions of rapid socioeconomic development in the southwestern region of China. Since China adopted the well-known “open-door” policy and economic reform, the near shore area of Lake Dianchi has experienced rapid industrialization and urbanization, which has resulted in regional ecosystem and environmental degradation, a loss of biodiversity, extreme deterioration of the water quality of Lake Dianchi, and other negative impacts [14,15,16,17]. Physical, chemical, biological, and systematic engineering methods and policies have been implemented to manage the environmental deterioration of the Lake Dianchi watershed [18,19,20,21]. However, the deteriorated status of the water quality and the environment have not been changed significantly, and the watershed continues to face multiple ecological crises [22,23,24]. The sole dependence on industrialized methods will make it difficult to fundamentally change the status of the water quality and environmental deterioration. Alternative approaches are needed to fundamentally address this problem. 

Some scholars have argued that the LULCC in the Lake Dianchi watershed should be studied for protecting the water quality, sustainable ecosystem and environment development [25]. Zhang *et al.* [26] analyzed the effect of LULCC structure on the watershed environment of Lake Dianchi. They found that the water quality near the Dianchi Lake drainage area is negatively affected by the developed area including town, village, factory, and mining construction. The larger the area is, the greater the effect on water quality pollution. They did not consider the land-use/land-cover pattern in their research. Tan *et al.* [27] simulated the process of urban sprawl of Kunming city from the perspective of ecosystem and environmental protection. However, few published reports have examined the spatial process and mechanisms of LULCC in the Dianchi watershed after the economic reform of China, which is a focus of the present study. The main objective of this study is to examine the spatiotemporal pattern and mechanisms of LULCC in the Lake Dianchi watershed following the economic reform by using remote sensing, GIS, and landscape metrics techniques. The results are expected to provide valuable information for assessing land-use policy and protecting the water quality and ecological well-being of the Dianchi watershed. 

## 2. Study Area

**Figure 1:**
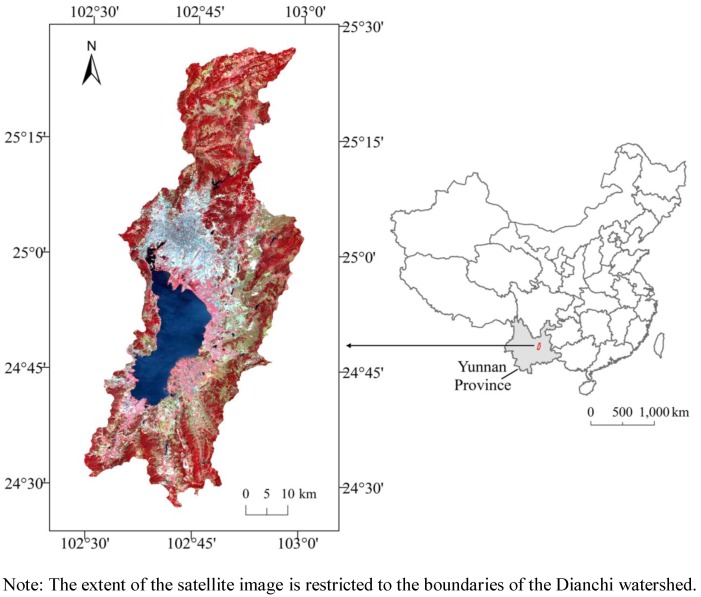
The Dianchi watershed and its location.

Lake Dianchi is located in the Yunnan-Guizhou Plateau of Southwest China in the upriver area of the Yangtze River (Figure 1). The Yunnan-Guizhou Plateau is one of four well-known Chinese plateaus (the other three plateaus are the Qinghai-Tibetan Plateau in the west, the Loess Plateau in the northwest, and the Inner Mongolia Plateau in the north). There are more than 30 large lakes in the Yunnan-Guizhou Plateau with water depths ranging from 10 to 50 m [28,29]. Lake Dianchi is the largest freshwater lake in the plateau and is the sixth largest body of freshwater in China; it is located in the central region to the southwest of Kunming, the capital of Yunnan Province. The geographic coordinates of Lake Dianchi are 102°29' to 103°01'E longitude and 24°29' to 25°28'N latitude, and the lake has an average altitude of 1,880 m. Lake Dianchi is nearly 40.4 km in length from north to south and 7 km from west to east with a total area of approximately 300 km^2^ and a total watershed area of approximately 2,834 km^2^. The urban areas of Kunming and the Jinning, Chenggong, Xishan, and Anning counties are located in the watershed. Generally, Dianchi is divided by an artificial causeway into two parts, Caohai and Waihai, occupying 2.7% and 97.3% of the lake surface area, respectively. The maximum and average depths are 6.5 m and 2.93 m, respectively. More than 20 rivers flow into Dianchi, which serves as the direct water source for the city of Kunming and the towns and villages around the lake. Tanglang is the only drainage river from Dianchi to Jinsha River (the upriver of the Yangtze River). The topography of the entire watershed slopes gradually from north to south. The mountains and lakes have a north-south trend due to the north-south geological fault zone in the watershed. The entire watershed is managed by the city of Kunming. In the Yunnan-Guizhou Plateau, Lake Dianchi plays an important role in regional water support, local micro-climate adjustment, the maintenance of biodiversity, and environmental protection of the Great Mekong Sub-region (GMS) [30].

The watershed has a mild temperate micro-climate with an annual mean temperature of 14.7 °C, as it lies in the wet monsoon climatic belt of northern subtropical zone. The warm climate and abundant rainfall are suitable for the growth of various terrestrial and aquatic species. The climate is characterized by a pronounced rainy season from May to October with an average annual rainfall of 947 mm and a dry season from November to April. The soil in the watershed is red mountain soil, and the vegetation is characterized by a subtropical, semi-humid, evergreen, broad-leaved forest. 

The Dianchi watershed area comprises only approximately 0.7% of Yunnan Province, although this region is responsible for 24% of the gross domestic product (GDP) of Yunnan Province. The Dianchi watershed is the most active economic area in Yunnan province [31]. Lake Dianchi serves many social and economic purposes. Most notably, it contributes to the water supply of Kunming, a city with a population of more than seven million people. The total watershed plays an important role in GMS cooperation. 

## 3. Methodology

### 3.1. Remote Sensing and GIS Techniques

Up until the launch of the first Earth Resources Technology Satellite (ERTS-1, later renamed Landsat 1) in 1972, LULCC studies have traditionally depended on survey data, aerial photographs and fieldwork. These approaches are expensive and very time-inefficient. Since the launch of the satellite, there has been significant activities related to mapping and monitoring LULCC as a function of anthropogenic pressures and natural processes [32,33]. Massive, multi-temporal, and remote sensing image databases from different sensors allow us to economically obtain information on regional land-use/land-cover patterns and change [32,34]. 

Remote sensing techniques have been widely applied for monitoring land-use/land-cover of watersheds and other relevant investigations. Prenzel *et al*. [35] demonstrated the efficacy of the hybrid change method for extracting thematic land-surface change information from satellite remote sensor data for a watershed in Indonesia. Xian *et al.* [36] utilized Landsat satellite images to map the urban extent and its changes for the Tampa Bay watershed of west-central Florida. Jat *et al.* [12] used remote sensing images acquired over eight years for the extraction of land-use/land-cover and urban growth data to assess the health of two urbanized sub-watersheds. Li *et al.* [37] used Landsat imagery to monitor the historic change in the lake water areas in the drought-affected Apalachicola-Chattahoochee-Flint (ACF) watershed in the US Several reports have proposed new remote sensing techniques for monitoring the land-use/land-cover dynamics of watersheds [38,39]. In short, these scholars extracted land-use/land-cover patterns and monitored their dynamics using remote sensing images and different classification approaches aided by GIS instead of traditional approaches. These works provide strong techniques for monitoring LULCC, exploring the mechanisms, and assessing the effect of LULCC on the environment. 

### 3.2. Data and Processing

This study adopts a series of Landsat images, including Landsat Multispectral Scanner (MSS) images with a spatial resolution of 57-m taken in January of 1974, and Landsat Thematic Mapper (TM) images with a spatial resolution of 30-m taken in January of 1988 and April of 1998 and 2008. A QuickBird image with 0.61-m spatial resolution acquired in March of 2008 was collected to cover the main urban area of Kunming as the reference data for the real region of interest (ROI), and for the extraction of land-use/land-cover patterns. All of the images were processed using geometric and radiometric corrections. Digital land-use maps from 1996 with vector structures and topographical maps from 1972 with a 1:50,000 scale also provided important information for identifying and assessing land use types for the time series images. 

Fieldwork was conducted in April and May of 2009 to acquire the direct observational data ROI required for the study. Before performing the fieldwork, the required amount of sample points was determined and the sample points were marked in the TM image. The sample points were intentionally selected to be close to the roads for the convenience of arriving and were numbered. The coordinates of the sample points were input into a portable Global Position System (GPS) receiver (with the V30 GNSS RTK system, produced by Guangzhou Hi-Target Survey Instruments Company Ltd, Guangzhou, China; http://www.zhdgps.com/en/index.aspx) for navigation. The Land-use/land-cover type of the sample point was investigated in detail in the fieldwork. 

Before China adopted the “open-door” policy and economic reform in 1978, the extent of industrialization was low, and few people migrated in or out of the Dianchi watershed. Therefore, the population of people and the land-use/land-cover pattern changed slowly. The Landsat MSS image from 1974 represents the land-use/land-cover pattern during the early stages of the Chinese economic reform in the late 1970s. Since 1988, Landsat images were collected at an interval of once every 10 years to monitor and analyze the LULCC of the Dianchi watershed after the adoption of the economic reform. The Landsat MSS image from 1974 was resampled to 30-m resolution to maintain the consistency of the image resolution throughout the time series. It is noted that no matter what technical processing that is done that an MSS image will never have the accuracy of a TM image 

### 3.3. Land-Use/Land-Cover Classification System

Classifying land-use/land-cover is one of the methods of understanding an environment to provide important information for national- or city- level plans for overcoming the problems of haphazard and uncontrolled development, deteriorating environmental quality, the loss of prime agricultural lands, the destruction of important wetlands, and the loss of fish and wildlife habitats [40]. Classification is a complex process that can be defined as “the ordering or arrangement of objects into groups or sets on the basis of relationships. These relationships can be based upon observable or inferred properties” [41]. Classification is related to the semantic meaning of the land use. Different studies may be so semantically different in the interpretation of the categorical meaning of land-use/land-cover that the results of classification become inconsistent with each other [42]. Existing land-use/land-cover classification systems are determined by the original purpose of the study and the data resource [39,43]. Some classification systems include multiple levels of classification [40], whereas others utilize a reduced number of classification types [44,45]. The use of too many or too few land-use/land-cover classification types affects the results of change analyses [43,46]. Various publications have discussed land-use/land-cover classification systems and have proposed appropriate classification systems for watershed research [35,39]. After considering the above discussion and the actual condition of the Dianchi watershed, a land-use/land-cover classification system was proposed for this study (Table 1). 

**Table 1:** Land-use/land-cover classification system of the Dianchi watershed.

Land-use/Land-cover Types	Description
Developed area	Urban area, rural developed area, transportation area, and industrial and mining areas
Forest land	Arboreal forest, shrubbery area, and economic forest
Wild grassland	Sparse woodland, rangelands in water deficit, and other grasslands
Water	Lakes, rivers, reservoirs, and ponds
Agricultural land	Irrigated arid land, unirrigated dry land, terraced land, vegetable land, and fruit land
Bare land	Bare rock, bare soil, vacant land, and other land that cannot be utilized

### 3.4. Land-Use/Land-Cover Classification Method

The supervised maximum likelihood method was used for the land-use/land-cover classification. A sufficient number of ROIs were selected in every image for different land-use/land-cover types, and the ability to differentiate between samples was tested (to ensure ROI separability > 1.80). Visual interpretation was then used to correct obviously mistaken land-use/land-cover types of certain pixels. The accuracy of the classification results were assessed using the total accuracy and the Kappa coefficient.

### 3.5. Dynamic Index of Land-Use/Land-Cover

The dynamic index of land-use/land-cover is used to quantitatively monitor the change in intensity of one land use type [47]. The dynamic index *K* is computed as follows:

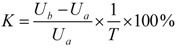
(1)

where *U_a_* and *U_b_* are the area of a certain land-use/land-cover type at the beginning and end of the research period, respectively. *T* is the length of time. When *T* is the year period, the value of *K* represents the change rate per year of a certain land-use/land-cover type. 

### 3.6 Landscape Metrics

Landscape metrics were developed in the late 1980s and incorporate measures from both information theory and fractal geometry based on a categorical, patch-based representation of the landscape. Patches are defined as homogenous regions for a specific landscape property of interest, such as “industrial land”, “park” or “high-density residential area”. Landscape metrics are used to quantify the spatial heterogeneity of individual patches, where all patches belong to a common class, and the landscape is a collection of patches. When applied to multi-scale or multi-temporal datasets, the metrics can be used to analyze and describe the changes in the degree of spatial heterogeneity [48,49,50]. 

The interest in using landscape metrics for the analysis of watersheds is beginning to grow. In 2001, Jones *et al.* [51] predicted nutrient and sediment loading to streams from landscape metrics for multiple watersheds in the United States Mid-Atlantic Region. Lundquist *et al.* [52] used landscape metrics to measure the suitability of a forested watershed. They proposed that landscape metrics may be useful in quantifying changes in landscape conditions as well as for monitoring forest planning in a watershed. Liu *et al.* [53] performed a study of landscape dynamics analysis using landscape metrics on a watershed scale. They examined the landscape dynamics of the Yashiro watershed in Japan at the landscape level using landscape metrics based on Landsat TM images from 1985 to 1998. Subsequently, landscape metrics were used to analyze the simulated and forecasted landscapes and land-use/land-cover in lake watersheds [54,55,56,57]. This study utilizes landscape metrics to explore the fragmentation, complexity, dispersion, and aggregation of the process of LULCC in the Dianchi watershed.

The number of patches (NP), mean patch size (MPS), edge density (ED), area-weighted mean shape (AWMS), perimeter-area fractal dimension (PAFRAC), and contagion index (CONTAG) of the landscape metrics were selected at the landscape scale. At the land-use/land-cover type scale, metrics of the largest patch index (LPI) and aggregation index (AI) were added to the above metrics. NP and MPS are used to represent the extent of fragmentation of a landscape. CONTAG and AI may reflect the degree of dispersion and aggregation of a landscape. ED, AWMS, and PAFRAC illustrate the complexity of a landscape. When the value of PAFRAC reaches 1.5, the landscape is in a stochastic status similar to that of Brownian motion, which is the most unstable status [58]. LPI is a measure of dominance and presents the extent of the aggregation of one land use category in the watershed. FRAGSTATS software is used to compute the value of these metrics. FRAGSTATS is a public domain spatial metrics program that was developed in the mid-1990s and has been continuously improved since its introduction [59]. FRAGSTATS provides a large variety of metrics at the class, patch and landscape levels. Table 2 describes the subset of available metrics used in this study. A more detailed description, including the specific mathematical equations for all of the metrics, can be found in McGarigal *et al*. [59].

**Table 2:** Landscape metrics used in this study.

Metric	Description	Unit	Range
NP: Number of patches	NP equals the number of patches of the corresponding patch type (class).	None	NP ≥ 1, no limit
MPS: Mean Patch Size	MPS equals the sum, across all patches in the landscape, of the corresponding patch metric values divided by the total number of patches and 10,000 (to convert into hectares).	Hectares per patch	MPS ≥ 0, no limit
ED: Edge Density	ED equals the sum of the lengths (m) of all edge segments in the landscape divided by the total landscape area (m^2^) multiplied by 10,000 (to convert into hectares).	Meters per hectare	ED ≥ 0, no limit
AWMS: Area-Weighted Mean Shape	AWMS equals the sum, across all patches in the landscape, of the corresponding patch metric value multiplied by the proportional abundance of the patch [ *i.e.*, the patch area (m^2^) divided by the sum of patch areas].	None	AWMS ≥ 1
PAFRAC: Perimeter Area Fractal Dimension	PAFRAC equals 2 divided by the slope of the regression line obtained by regressing the logarithm of the patch area (m^2^) against the logarithm of the patch perimeter (m).	None	1 ≤ PAFRAC ≤ 2
CONTAG: Contagion Index	CONTAG measures the overall probability that a cell of a patch type is adjacent to cells of the same type.	Percent	0 < CONTAG ≤ 100
AI: Aggregation Index	AI equals the number of like adjacencies involving the corresponding class divided by the maximum possible number of like adjacencies involving the corresponding class, which is achieved when the class is maximally clumped into a single, compact patch; multiplied by 100 (to convert into a percentage).	Percent	0 < CONTAG ≤ 100
LPI: Largest Patch Index	LPI equals the area (m^2^) of the largest patch of the corresponding patch type divided by the total landscape area (m^2^) multiplied by 100 (to convert into a percentage); *i.e.*, LPI equals the percentage of the landscape comprised by the largest patch.	Percent	0 < LPI ≤ 100

## 4. Results and Discussion

### 4.1. Land-Use/Land-Cover Maps and Accuracy Assessment

**Figure 2:**
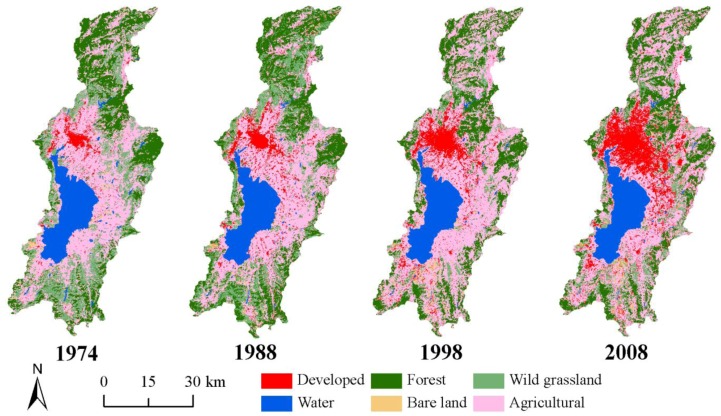
Land-use/Land-cover maps in 1974, 1988, 1998, and 2008.

Land-use/land-cover classification maps of the Dianchi watershed for 1974, 1988, 1998, and 2008 were produced (Figure 2). The classification results were assessed using a confusion matrix based on real ROI data obtained via fieldwork and high-resolution images. The results reveal that the total accuracies of the land-use/land-cover classification were 78.85%, 88.94%, 92.36%, and 94.44% for 1974, 1988, 1998, and 2008, respectively, and the Kappa coefficients for these years were 0.73, 0.85, 0.89, and 0.93, respectively. Because the resolution of the Landsat MSS images acquired in 1974 is 57-m, these images were resampled to a 30-m resolution to maintain the consistency of the image resolution, and thus the total accuracy and the Kappa coefficient for 1974 are a little lower than for the other years. Generally, the accuracy is sufficient to meet the monitoring needs of the LULCC in the Dianchi watershed.

Intuitively, Lake Dianchi is surrounded by agricultural lands in the alluvial plains which adjacent to the north, east, and south of Lake Dianchi. Agricultural land is also found to the west of the lake. However, due to the effects of the topography, the area of agricultural land to the west of the lake is relatively small. The urban area of Kunming is located in the middle-northern area of the northern plain of the Dianchi watershed. There are also dispersed developed areas in other plains of the watershed. Forests and wild grasslands are primarily located in mountainous areas around the alluvial plains. A large escarpment occurs in the northwest of Lake Dianchi. Therefore, this part of Lake Dianchi is almost directly connected to mountains covered with forest and wild grassland, and no plain buffer area exists between the lake and the mountain. Obviously, the land-use/land-cover pattern is related to the geographical conditions of the Dianchi watershed. 

In the time series, the LULCC during the period of 1974–2008 was markedly characterized by the expansion of the urban area of Kunming and changes in the agricultural land pattern. The urban area expanded into much of the agricultural land, and the agricultural land expanded into the north and south of the watershed. Other land-use/land-cover types did not display such intuitive and obvious changes and appeared to be occupied by agricultural land.

### 4.2. Structure, LULCC, and Landscape

Table 3 illustrates the land-use/land-cover structure and its change in the Dianchi watershed over a four-time-point series. Agricultural land is the primary land-use/land-cover type in the land-use/land-cover structure and accounted for more than 40% of the total watershed at all four of the time points in the series, which is approximately half of the total study area, implying that agriculture has played an important role in the socioeconomic development of the watershed. The relative change rate of agricultural land during 1974–2008 was only 1%, indicating little change in the agricultural land area. Agricultural land change is characterized by its pattern and distribution (Figure 2). Forest land following agricultural land in the area accounted for a constant 22% to 26% of the total watershed, and the relative rate of change was only 2.8% from 1974 to 2008. In 1974, wild grassland was ranked third at 18.8% of the total watershed area. By 2008, however, the proportion of wild grassland decreased to 7.1% with a decrease in area of approximately 331.4 km^2^ and a relative change rate of −62.1%. The water area was ranked fourth, and its area decreased by approximately 22.8 km^2^ from 1974 to 1988. Subsequently, the proportion of water remained at 10.9% with little change in the area. Bare land occupied very little of the total watershed area, at approximately 1.5%, and did not show a substantial change. The developed area changed the most out of all six of the land-use/land-cover types, increasing from 98.4 km^2^ in 1974 to 319.2 km^2 ^to 417.6 km^2^ in 2008. The proportion of the entire watershed area attributed to developed areas changed from 3.5% in 1974 to 14.7% in 2008 with a relative rate of change of 324.4%; this land type increased from ranking fifth in 1974 to third in 2008. 

**Table 3:** Structure and changes in land-use/land-cover: 1974, 1988, 1998, and 2008.

Land-Use/Land-Cover Type	1974		1988		1998		2008		Relative Change 1974–2008 (%)
	Area (km^2^)	%	Area (km^2^)	%	Area (km^2^)	%	Area (km^2^)	%	
Developed area	98.4	3.5	178.4	6.3	244.4	8.6	417.6	14.7	324.4
Agricultural land	1,175.8	41.5	1,151.7	40.6	1,399.4	49.4	1,187.6	41.9	1.0
Forest land	648.3	22.9	714.1	25.2	671.9	23.7	666.8	23.5	2.8
Wild grassland	533.5	18.8	440.7	15.5	163.4	5.8	202.1	7.1	−62.1
Bare land	46.6	1.6	40.5	1.4	48.7	1.7	50.4	1.8	8.2
Water	332.2	11.7	309.4	10.9	307.1	10.8	310.2	10.9	−6.6

We calculated the transition area of the land-use/land-cover types from 1974 to 2008 (Table 4). Table 4 illustrates that new developed area in 2008 was mostly derived from agricultural land, whereas new agricultural land from forest land and wild grassland, new forest land from agricultural land and wild grassland, wild grass land from agricultural land, and bare land from agricultural land and wild grassland. 

The changes in land-use/land-cover in the Dianchi watershed are related to the rapid urbanization process in China in the 1970s. Urbanization became an important theme in socioeconomic development after 1978 when China adopted the “open door” policy and economic reform [60]. The Dianchi watershed also experienced such a rapid urbanization process [14,61].

**Table 4:** Transition matrix of land-use/land-cover types from 1974 to 2008 (Unit: km^2^).

1974/2008	Developed Area	Agricultural Land	Forest Land	Wild Grassland	Water	Bare Land	Total
Developed area	66.9	25.1	2.3	2.6	0.7	0.8	98.4
Agricultural land	291.4	673.0	119.5	66.2	3.2	22.5	1,175.8
Forest land	10.1	194.9	418.3	21.4	0.6	2.9	648.3
Wild grassland	38.5	249.8	119.4	103.1	3.0	19.8	533.5
Water	4.6	22.9	0.7	0.7	302.6	0.8	332.2
Bare land	6.1	22.0	6.7	8.1	0.1	3.7	46.6
Total	417.6	1,187.6	666.8	202.1	310.2	50.4	2,834.8

The rapid urbanization process was accompanied by the transfer of a large number of people from rural to urban areas, the rapid development of the real estate industry, and the extension of transportation network, green space in urban area, and other urban infrastructure. The rapid increase in the urban population was also accompanied by rapid growth in the demand for food, vegetables, fruit and other agricultural production as well as demand for housing, drinking water and other infrastructure. The great expansion of the developed area of the Dianchi watershed between 1974 and 2008 intuitively represents the rapid urbanization process in the land-use/land-cover pattern. The expansion of the urban area is characterized by a mode of urban sprawl into surrounding agricultural land, which is similar to the urbanization processes in other regions of China [62]. The decrease in the water area may be closely related to the water demands of urban residents. However, the use of land for agricultural purposes did not decrease during the period between 1974–1998, implying that the important role of agricultural land was not curtailed, and food security was provided to ensure the sustainability of human life in the Dianchi watershed.

**Figure 3:**
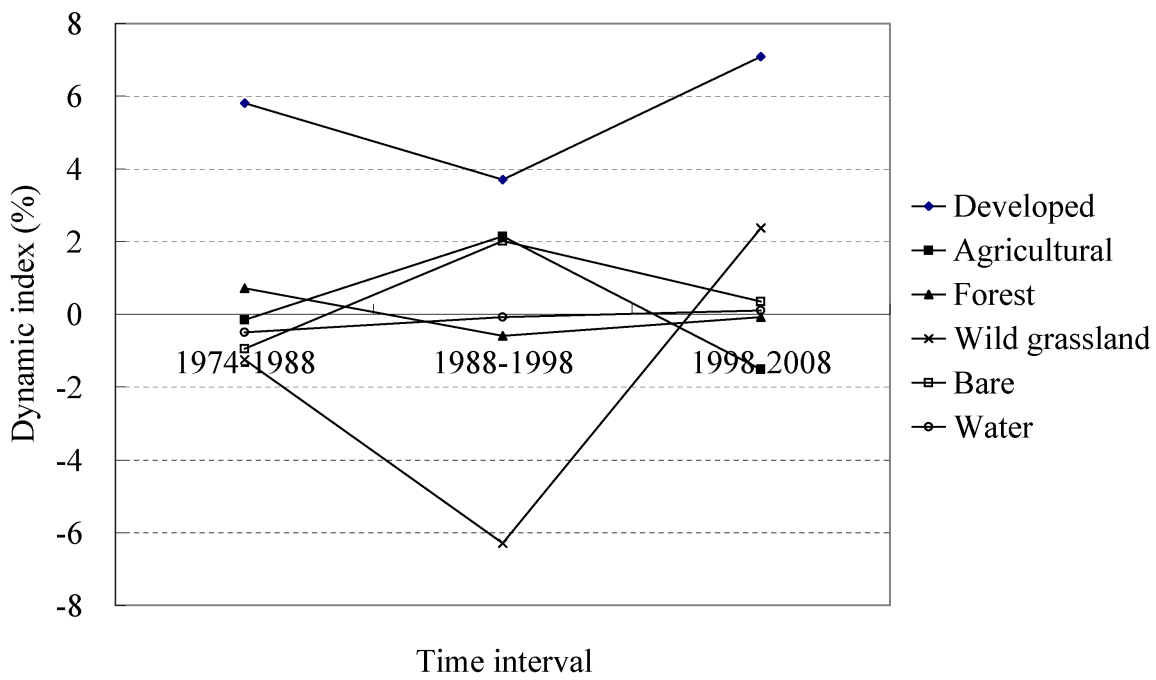
Dynamic index of land-use/land-cover at the class level.

Figure 3 indicates the dynamic index of the LULCC. The dynamic index of the developed area is the largest out of the six land-use/land-cover types and illustrates the characteristics of rapid expansion in the developed area in the LULCC of the watershed. The dynamic index of wild grassland changed greatly from 1974 to 2008; the value of the index was −1.2% during the period of 1974 to 1988 and decreased to −6.3% during the period of 1988 to 1998. However, the index increased to 2.4% during 1998–2008. Wild grassland includes the sparse woodland, and thus, the increase in the wild grassland may have been derived from agricultural lands as a result of the “farmland (with a slope greater than 25°) returns to woodland” policy of China. Table 4 illustrates that from 1974 to 2008 approximately 66.2 km^2^ of agricultural land was converted into wild grassland, and 119.5 km^2^ into forest land. Dynamic indices of other land-use/land-cover types changed little and remained within the interval of −2.0% to 2.0%.

**Figure 4:**
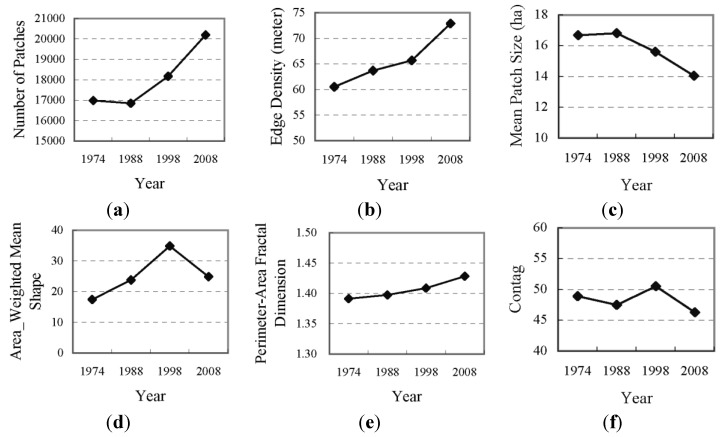
Metrics of land-use/land-cover at the landscape level.

The landscape metrics shown in Figure 4 indicate the dynamics of the spatial pattern and the shape of the Dianchi watershed landscape from 1974 to 2008. The NP value changed little from 1974 to 1988 and maintained an increasing trend since 1988, strongly illustrating the spatial heterogeneity of the development trend and fragmentation of the total landscape. Accordingly, the MPS value did not change appreciably from 1974 to 1988, but decreased after 1988, confirming the trend of spatial heterogeneity and landscape fragmentation. The ED value continued to increase during the study period. Although the NP value changed little from 1974–1988, the ED value increased, thereby indicating the enhancement of edge complexity. The ED value increased greatly from 1998 to 2008. This large change may be a result of the rapid increase of NP during this period. The AWMS value continued to increase from 1974–1998, indicating an enhancement of the non-regularity of patches in the watershed. However, the value decreased after 1998, illustrating the gradual regularization of the shape of patches. The PAFRAC value continued to increase during the period and approached the unstable status point of 1.5, thereby implying a decrease in the stability of the land-use/land-cover structure of the landscape in the Dianchi watershed. The landscape of the watershed displays a dynamic trend. The CONTAG analysis revealed a series of fluctuating values. These values decreased slightly from 1974–1988, and after 1988, they rose suddenly to a maximum value of 50.51. This value then decreased to below the maximum value in 1974. The dynamics of the CONTAG index were similar to the changes in the agricultural land in the land-use/land-cover structure shown in Table 3. Former agricultural land was mostly developed into built-up areas, while the new increased agricultural land was derived from wild grassland. Therefore, the CONTAG index illustrates the relationships of patch shape and contagion degree between the primary land-use/land-cover type of agricultural land, the markedly changed developed area, and the wild grassland. 

Scale is an inherent landscape attribute in LULCC observations [63]. It is noted that the landscape metrics used in this study, although scale dependent [64], were calculated based on 30-m resolution.

### 4.3. Developed Area Changes

**Figure 5:**
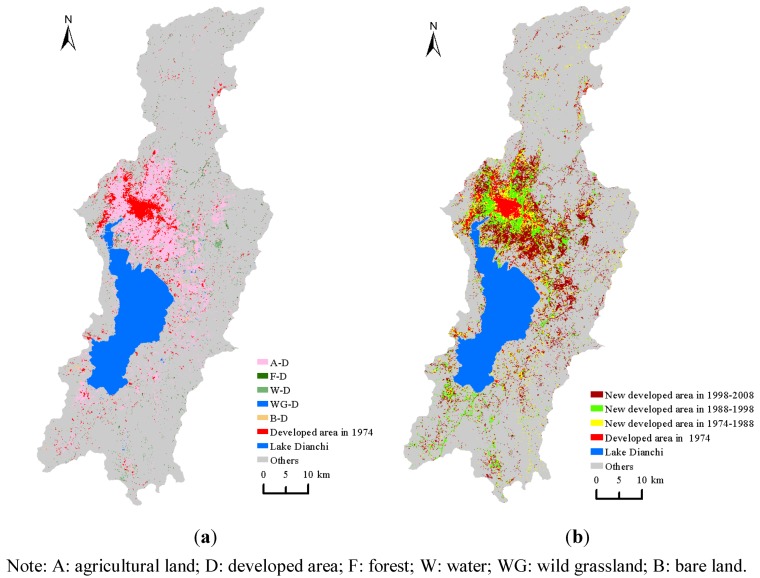
Urban sprawl dynamics in the studied time intervals (**a**) and the transformation of land use types to developed areas from 1974 to 2008 (**b**).

The expansion of developed areas in the three time intervals of 1974–1988, 1988–1998, and 1998–2008 were computed (Figure 5(a)). The dynamics of the developed area in the Dianchi watershed are influenced by the urban sprawl of the unique main city of Kunming and assume the typical expansion mode of cyclical structures and jigsaw patterns. The expansion of the developed area in other regions was more dispersed. As a whole, new developed area consistently appeared near existing developed areas in the studied time intervals and typically expanded in a cyclical pattern. In addition, due to the irregularity and emergence of existing developed areas, newly built areas expanded outward while constantly and gradually filling the vacant land areas adjacent to the existing developed area. This expansion of a new developed area and an existing developed area formed a jigsaw pattern. In the transformation of land-use/land-cover type, the expansion of the developed area primarily occupied agricultural land (Figure 5(b)) and expanded to the shore of Lake Dianchi. The space for expansion was very limited for both agricultural land and developed areas due to the narrow topography of the watershed; this was especially true for the spatial development, as natural conditions have limited the room for expansion. Due to this limitation, the new developed areas must occupy the agricultural land surrounding the urban area of Kunming and other old cities, which has led to a large decrease in agricultural land. 

The changes in the developed area in the watershed correlate with urban land reform in China. In the late 1970s, China began rural and urban land reforms [65,66]. The effect of these reforms, especially the urban land reform, has caused purely administrative allocation practices to become gradually replaced by the commercialization of urban land use rights in all cities.

In addition, China’s fiscal system began a tax division reform in 1994, which divided taxes into those levied by the central government, local taxes, and taxes shared between the State and local governments. Local governments may collect three types of tax: an income tax on local business, an urban land use tax, and a land appreciation tax. Such urban land use and fiscal reforms have been enthusiastically received by city governments [66]. These reforms caused two problems. First, local governments established many development zones for the development of industrial enterprises on cheaply acquired agricultural land to increase local tax revenue. Land acquisition is one administrative mode of land transformation from agricultural lands to developed areas [67]. To attract additional industrial capital, the land transfer fee was set at extremely low levels or was free. In Kunming, the Kunming National Hi-tech Industries Development Zone, Kunming Economic and Technological Development Zone, and other zones were established in 1980s. These development zones are directly managed by the Kunming municipality. Second, land for residential and commercial use was transferred through market mechanisms to enable the city government to extract further income from land transfers. These revenues were then typically used for infrastructure investment in other areas, a practice known as “financing the city”. With the rapid growth of the urban economy, large numbers of migrant workers swarmed into the city, further increasing the price of residential and commercial land. With a decrease in available state-owned land and an increase in urban redevelopment costs, collectively owned rural land inevitably became a target for requisition. The implementation of these urban land-use policies and regulations has had a positive impact on local socioeconomic development. However, these policies and regulations have directly caused the rapid expansion of the urban area of Kunming into the near shore area of Lake Dianchi. This greatly increased developed area has brought more household and industry waste to Lake Dianchi, with serious consequences for water quality and environmental pollution more serious [31,61]. Therefore, new urban land-use policy and regulation should be constituted to limit the rapid progress of urban sprawl in the near shore area of Lake Dianchi.

**Figure 6:**
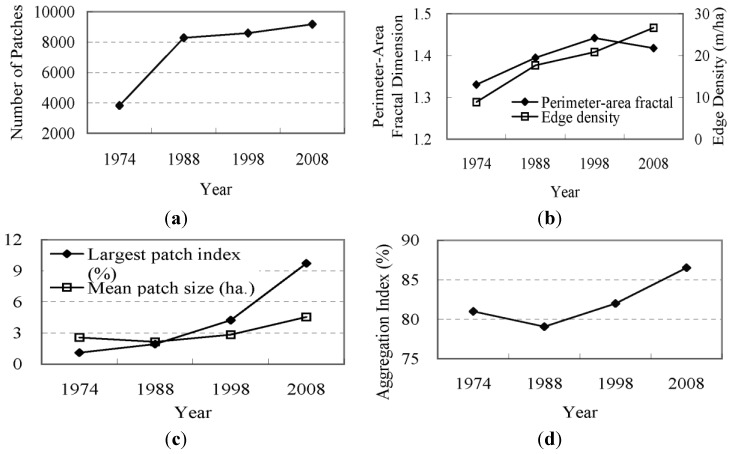
Dynamics of the landscape metrics of developed areas.

Here, we examine the change in shape of the developed area. Figure 6 illustrates the dynamics of the landscape metrics of the developed area from 1974–2008. The NP value increased greatly from 1974–1988 and leveled off to a slower, steady growth level from 1988 onward (Figure 6(a)). 

Correspondingly, continuous growth in the ED value was also observed from 1974–2008 (Figure 6(b)). According to the dynamic index of the developed area displayed in Figure 2, the increases in the NP and ED values illustrate an increase in the number of developed patches and an enhancement of the edge complexity. The reduction of the MPS in the period from 1974 to 1988 (Figure 6(c)) indicates that new developed areas primarily emerged in other dispersed regions outside of the city and reveals the emergence and dispersion of new developed areas. Newer developed areas that were developed since 1988 remained concentrated in the region near the main urban area and assumed the characteristics of convergence. The curve of the AI value (Figure 6(d)) and the increase of LPI (Figure 6(c)) confirm this finding. The PAFRAC value maintained a continuous increase during the period of 1974–1998 and eventually reached the most unstable status point of 1.5. This value has decreased since 1998. These results may represent the characteristics of change in the developed area of the watershed. Between 1974 and 1998, urban sprawl was characterized by a mode of expansion that spread out from the old town center to the surrounding agricultural land. With the convergence of the newly developed areas, the overall shape of the developed area became more complex. During 1998–2008, a large number of simple patches of new developed area emerged amid the rapid urban sprawl.

### 4.4. Agricultural Land Changes

**Figure 7:**
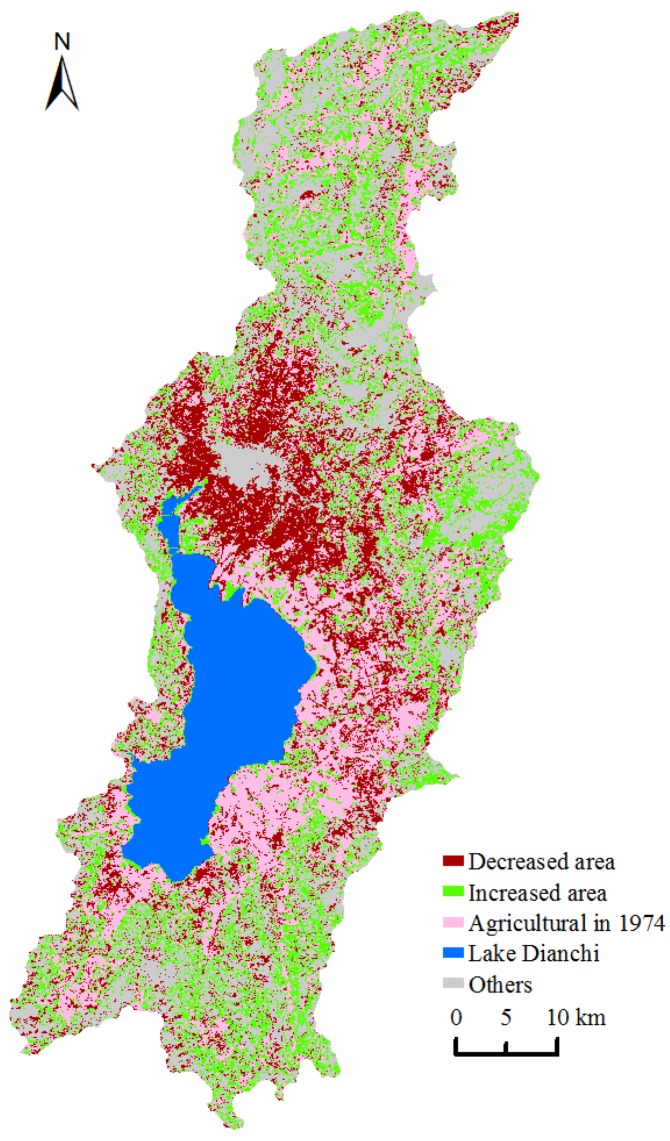
Transformation into and out of agricultural land from 1974–2008.

There was no obvious change in the area of agricultural land during 1974–2008. However, as the large expansion in newly developed land occupied much of the agricultural land during this period, it is deduced that the shape and spatial pattern of agricultural land has changed greatly. Land-use/land-cover maps of the four time intervals shown in Figure 2 provide an intuitive illustration of this effect. Figure 7 indicates the spatial pattern of transformation into and out of agricultural land from 1974–2008. From this pattern, we can see that the reductions in agricultural land were used primarily for transformation into developed areas, and the increases in new agricultural land were distributed widely throughout the regions that were previously wild grasslands and forest land. 

In China, expansions of developed areas tend to occupy agricultural lands surrounding urban areas. To address the rate of decrease and reduced quality of agricultural lands, the central government of China implemented a dynamic equilibrium and dynamic balance policy for the total amount of cultivated land (*i.e.*, cultivated land expropriation must be compensated by reclaimed land with equivalent qualities in another place) [68,69]. Therefore, although new developed areas occupied a large area of agricultural land surrounding the urban area of the Dianchi watershed during the period of 1974–2008, this occupied agricultural land was complemented in other regions of the watershed by the implementation of the dynamic equilibrium and dynamic balance policy regarding the total amount of cultivated land. This practice is likely the primary reason why there has been no obvious change in the structure of agricultural land in the Dianchi watershed. However, because newly increased agricultural land was mostly derived from forest land and wild grassland in the watershed (Table 4), the dynamic equilibrium and dynamic balance policy had, to a certain extent, a negative impact on the ecosystem and environment of the Dianchi watershed. Therefore, limiting rapid urban sprawl is an important step that should be implemented to avoid ecosystem and environmental degradation in the watershed.

**Figure 8:**
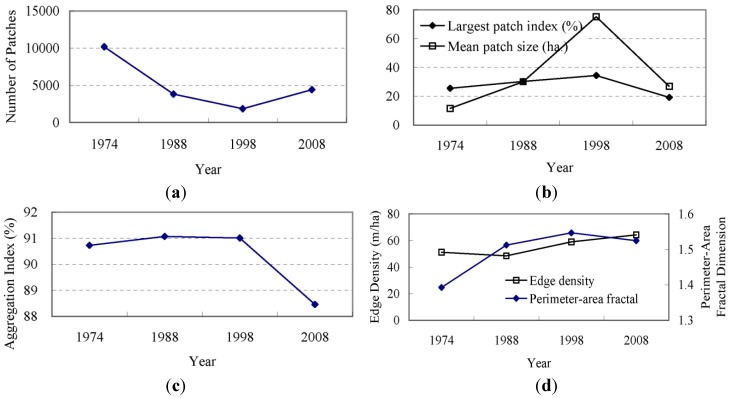
Dynamics of the landscape metrics of agricultural lands.

The NP value of the agricultural land decreased continuously from 1974–1998 and then slightly increased after 1998 (Figure 8). The MPS value gradually increased during 1974–1998 and then significantly decreased. This phenomenon indicates the convergence of agricultural land during the period of 1974–1998 as well as the dispersion trend after this time. 

The subsequent decline of the LPI and AI after 1998, *i.e.*, after these values had experienced gradual increases during 1974–1998, also confirms this finding. The ED value decreased from 1974 to 1998, indicating an enhancement of the edge regularity of agricultural land patches. This value gradually increased after 1998, meaning that the boundaries of the agricultural lands became more complex. Large-scale agricultural lands in the near shore areas of Lake Dianchi were divided into small patches by the new developed area during the expansion of the urban area. This made the boundary of the agricultural lands more complex, which was consistent with the increasing complexity of the entire landscape of the watershed. The PAFRAC value showed a continuous increase from 1974 to 1998, reflected the increasing complexity of the agricultural land patches. Although the PAFRAC value decreased after 1998, it is similar to the most unstable status point of 1.5, indicating the growing instability in the structure of agricultural land.

## 5. Conclusions

This paper examined the spatiotemporal land-use/land-cover pattern in the Lake Dianchi watershed of the Yunnan-Guizhou Plateau of Southwest China at four time points, 1974, 1988, 1998, and 2008, using Landsat MSS/TM images with remote sensing and GIS techniques. The land-use/land-cover pattern and its changes in structure, shape, and distribution were analyzed, and the mechanism of the changes was discussed using post-classification overlay and time series analysis approaches with landscape metrics. 

The land-use/land-cover of the Dianchi watershed was classified into six types, *i.e.*, developed area, agricultural land, forest land, wild grassland, bare land, and water. Lake Dianchi is surrounded by agricultural lands in the alluvial plains, which are adjacent to the north, east, and south of Lake Dianchi. Agricultural land accounts for more than 40% of the land area the total watershed, implying that agriculture has played an important role in the socioeconomic development of the watershed. The developed area is mostly located in the north central area of the northern alluvial plain. Forests and wild grasslands are primarily located in mountainous areas around the alluvial plains. The land-use/land-cover pattern is related to the geographical conditions of the Dianchi watershed. 

The developed area showed the greatest change in the land-use/land-cover type in the Dianchi watershed from 1974–2008; this area increased by 319.2 km^2^ from 98.4 km^2^ in 1974 to 417.6 km^2^ in 2008, with a relative change rate of 324.4% during the 34 years from 1974 to 2008. In terms of changes in the spatial pattern, the dynamics of the developed area were characterized by the urban sprawl of Kunming with a typical cyclical structure and jigsaw pattern model of expansion. The expansion of the developed area in other regions was more dispersed. Expansions in the new developed area predominantly occupied agricultural land surrounding urban areas, and the shape of this expansion revealed a land use pattern that became increasingly complex. The rapid expansion of the developed area was a microcosm and manifestation of the rapid urbanization process of China after China adopted the “open door” policy and economic reform, and the expansion was closely related to the urban land use reform of China. The large increase in the developed area was the primary driving force of ecological environment degradation in the Dianchi watershed. 

Agricultural land is the primary land-use/land-cover type in the Dianchi watershed and occupies greater than 40% of the total area of the watershed. With a low relative change rate of 1%, there were very few changes in the land use structure of agricultural land during the period of 1974–2008; however, the agricultural land use pattern was substantially changed. Agricultural lands surrounding urban areas were transformed into developed areas, and other land-use/land-cover types, such as wild grasslands, were transformed into agricultural land according to the dynamic equilibrium and dynamic balance policy regarding the total amount of cultivated land in China. The dynamic equilibrium and dynamic balance policy brought negative impact on ecosystem and environment of the Dianchi watershed at a certain extent as new cultivated agricultural land mostly occupied forest land and wild grassland. The shape of the agricultural land also developed complex changes. The change in the pattern and shape of agricultural land use was also closely related to the demand of agricultural land as an urban hinterland, the rapid urbanization process and the increase in population. 

New urban land-use policy and regulation should be instituted to limit the rapid progress of urban sprawl in the near shore area of Lake Dianchi to protect water quality from household and industry waste pollution and avoid the ecosystem and environmental degradation in the watershed. The land-use policy decision-making from the perspective of the water quality and environmental protection for the Dianchi watershed would be a valuable extension to this research. We will concentrate on this issue by using geo-simulation method in the next step.
